# Clusterin: a double-edged sword in cancer and neurological disorders

**DOI:** 10.17179/excli2024-7369

**Published:** 2024-07-09

**Authors:** Pinky Sultana, Jiri Novotny

**Affiliations:** 1 Department of Physiology, Faculty of Science, Charles University, Prague 128 00, Czechia; 2 Laboratory of Genome Integrity, Institute of Molecular Genetics of the Czech Academy of Sciences, Prague 142 20, Czechia

**Keywords:** aging, cancer, cellular senescence, clusterin

## Abstract

Clusterin is a ubiquitously expressed glycoprotein that is involved in a whole range of biological processes. This protein is known to promote tumor survival and resistance to therapy in cancer, which contrasts sharply with its neuroprotective functions in various neurological diseases. This duality has led to recent investigations into the potential therapeutic applications of clusterin inhibition, particularly in cancer treatment. Inhibition of clusterin has been shown to be able to induce cancer cell senescence, suppress their growth and increase their sensitivity to therapy. The involvement of clusterin in the aging process makes its biological effects even more complex and offers a broad perspective for research and therapeutic exploration of various pathological conditions. This review critically examines the multiple functions of clusterin in cancer and neurological disorders and addresses the controversies surrounding its role in these areas. The assessment includes an in-depth analysis of the existing literature and examining the relationship of clusterin to fundamental aspects of cancer progression, including cell proliferation, apoptosis, metastasis, and drug resistance. In addition, the review addresses the neurobiological implications of clusterin and examines its controversial role in neuroprotection, neurodegeneration, and synaptic plasticity. Attention is also paid to the epigenetic regulation of clusterin expression. By clarifying conflicting findings and discrepancies in the literature, this review aims to provide a nuanced understanding of the molecular mechanisms underlying clusterin functions and its potential clinical implications in both cancer and neurodisorders.

See also the graphical abstract(Fig. 1).

## Introduction

Clusterin (CLU), also known as apolipoprotein J (ApoJ), is a secreted glycoprotein with a highly conserved heterodimeric structure. It is expressed in a wide range of tissues and can be detected in all human body fluids (Jones and Jomary, 2002[71]). This protein has been found to be associated with a variety of physiological processes, including sperm maturation, lipid transport, regulation of the complement system, tissue remodeling, cell interactions, membrane recycling, stabilization of proteins under stress, and promotion or inhibition of apoptosis. Remarkably, the ApoJ gene is expressed at different levels of expression in response to cytokines, growth factors, and stress-inducing agents (Trougakos and Gonos, 2002[166]). A crucial and interesting feature of CLU is its upregulation in severe physiological disorders and various neurodegenerative diseases that are often associated with advanced aging. In addition, CLU accumulates during the state of viable growth arrest known as senescence, which is thought to contribute to the aging process and the suppression of tumorigenesis (Trougakos and Gonos, 2002[166]). Paradoxically, CLU has also been observed to be upregulated in certain cases of cancer progression and tumor formation (Tellez et al., 2016[159]). The dual nature of CLU presents a challenge and an opportunity for therapeutic intervention. Understanding the intricacies of its involvement in cancer and neurodegenerative diseases is crucial for the development of targeted therapies. Researchers are investigating the possibility of modulating CLU levels or CLU activity to harness the protective aspects of CLU while mitigating its potential toxic effects. Given the role CLU plays in age-related processes, deciphering CLU functions may be key to developing interventions that combat certain diseases and promote healthier aging. The multifaceted nature of CLU adds to the complexity to the ongoing debate and calls for continued exploration and nuanced approaches in therapeutic development.

## Discovery of Clusterin and Basic Characteristics

**Figure 2:**
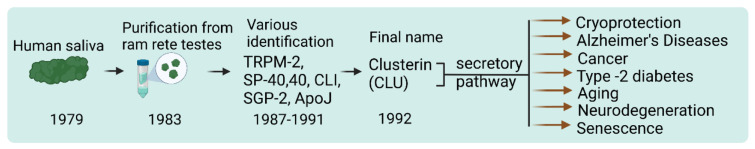
Illustration of the discovery and purification process of clusterin. The first steps include the identification and isolation of the protein, followed by purification and identification of its biological role.

CLU was originally discovered in 1979 from human saliva that showed aggregating properties towards streptococcal strains (Hogg and Embery, 1979[62]). CLU was then purified by chromatography from ram testicular fluid in 1983 and identified as an 80-85 kD sulfated glycoprotein that causes aggregation (Blaschuk et al., 1983[10], Fritz et al., 1983[47]). Over the years, CLU was given several names, such as testosterone-repressed prostate messenger-2 (TRPM-2) (Leger et al., 1987[85]), serum protein (SP-40,40) (Murphy et al., 1988[109]), complement lysis inhibitor (CLI) (Jenne and Tschopp, 1989[66]), sulfated glycoprotein ( SGP-2) (Purrello et al., 1991[130]), apolipoprotein J (apoJ) (de Silva et al., 1990[33]). Later, the term 'clusterin' was coined during the inaugural CLU workshop in 1992 in Cambridge, UK (Fritz and Murphy, 1993[48]). Subsequently, several homologs were isolated/cloned and named according to their origin. A brief overview of the history of CLU can be found in Figure 2(Fig. 2).

### Clusterin expression

CLU is present in diverse cell types, including specialized secretory and epithelial cells (Aronow et al., 1993[3]). In the brain, mainly astrocytes produce CLU, but neurons also partially take up CLU from the extracellular space (Pasinetti et al., 1994[124]). CLU accumulates in cortical neurons of aged and type-2 diabetic mice (Liu et al., 2022[92]). CLU can stabilize cell membranes at fluid-tissue interfaces and thus may play a role in barrier cytoprotection (Aronow et al., 1993[3]; Londou et al., 2008[94]). Higher CLU expression was found to be associated with an increase in programmed cell death, suggesting its role in cell survival (Bandyk et al., 1990[5]; Jeong et al., 2014[68]). Importantly, increased CLU levels have been found in cancer and Alzheimer's disease patients (Bertrand et al., 1995[9]; Matsubara et al., 1996[102]; Xiu et al., 2015[183]). Interestingly, CLU can bind to the Alzheimer's disease-related peptide amyloid β (Aβ) and effectively inhibit its aggregation (Bertrand et al., 1995[9]; Matsubara et al., 1996[102]; Xiu et al., 2015[183]). The data obtained so far indicate that the upregulation of CLU could be one of the main risk factors for aging, cancer, and neurodegenerative disorders.

### Clusterin synthesis and isoforms

**Figure 3:**
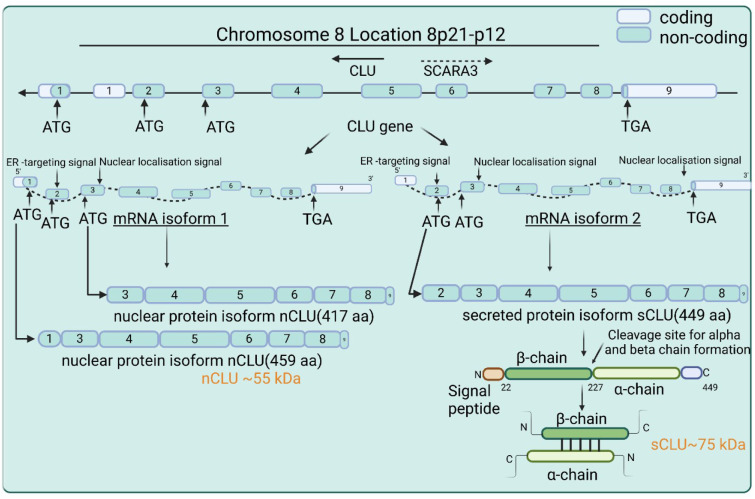
Schematic representation of the clusterin gene and its isoforms. The CLU gene, located on chromosome 8 at the p21-p12 locus, gives rise to several isoforms, including secretory (sCLU) and nuclear (nCLU) isoforms. The process involves transcription, alternative splicing, and translation, resulting in protein product with diverse cellular functions.

CLU is a conserved single-copy gene situated on chromosome 8 within the p21-p12 locus (Wong et al., 1994[180]). It is abundant in plasma and cerebrospinal fluid (Schrijvers et al., 2011[146]; Jongbloed et al., 2014[72]). It consists of nine exons encoding a 2.8 kb mRNA and is translated into a primary polypeptide chain of 449 amino acids (Jones and Jomary, 2002[71]). Exon 2 contains the initiation codon, producing a preprotein with an N-terminal ER-signal peptide. This peptide is cleaved during ER translocation to enable synthesis of the secreted protein. Following N-glycosylation in the ER, the pre-secretory CLU relocates to the Golgi for further processing. Cleavage in the Golgi yields alpha and beta chains linked by 60 kDa disulfide bonds (Urban et al., 1987[169]). Further glycosylation leads to the formation of a mature secretory CLU protein of 75-80 kDa. Secretion of CLU generally occurs from vesicles via the secretory pathway; uptake and degradation are likely mediated by the endocytic receptor LRP-2 from the low density lipoprotein receptor gene family (Kounnas et al., 1995[80]). Conversely, exon 3 and exon 8-9 contain nuclear localization signals. The alternative isoform is post-translationally modified to form a 55 kDa pro-apoptotic nuclear CLU protein (Figure 3(Fig. 3)). Regulation of nuclear clusterin (nCLU) expression in irradiated cells involves both nuclear localization and export sequences (NESs) that affect cell death/survival signaling. Moreover, mutation of the C-terminal nuclear localization signal (NLS) impairs the function of nCLU (Leskov et al., 2003[88]). The sCLU/nCLU ratio has a significant impact on how cancer cells respond to genotoxic and ionizing radiation and influences proliferation and metastasis in prostate cancer. The use of an "on-demand alternative splicing" method to reduce this ratio could improve therapeutic outcomes by inducing apoptosis in prostate cancer cells during chemotherapy and radiotherapy (Essabbani et al., 2013[41]).

## Clusterin in Cancer

### The involvement of CLU in the molecular mechanism of tumorigenesis

**Figure 4:**
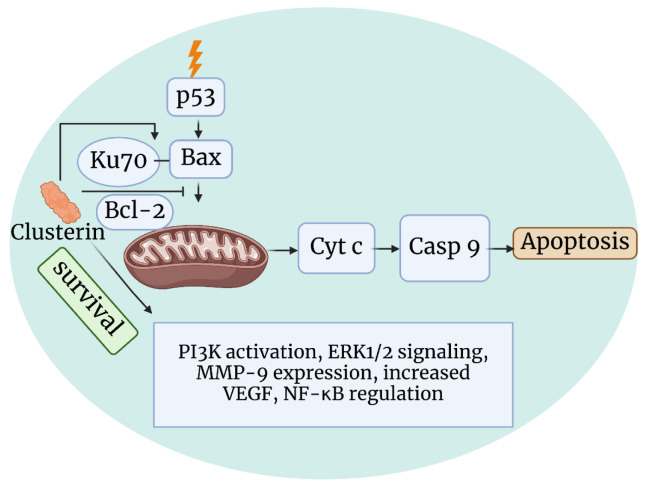
Illustration showing the anti-apoptotic mechanism of clusterin by binding to Ku70. In response to apoptotic signals, CLU interacts with the Ku70 protein and forms a complex that inhibits the apoptotic cascade. This interaction may involve the sequestration of Ku70, preventing its involvement in pro-apoptotic pathways. The figure highlights the molecular interplay between CLU and Ku70 that contributes to the anti-apoptotic function of CLU.

The importance of CLU is widely recognized in the scientific community as it is critically involved in various biological processes such as cell survival, apoptosis, metastasis, and chemoresistance. CLU is overexpressed in several types of cancer, including prostate cancer (July et al., 2002[73]), breast cancer (Ranney et al., 2007[132]), and lung cancer (Panico et al., 2009[120]). Initial findings suggested that cytoplasmic CLU (isoform 2) supports cell survival and has a cytoprotective effect (Poon et al., 2000[126]), while nCLU (isoform 1) plays a more pro-apoptotic role (Leskov et al., 2011[87]; Rizzi et al., 2009[137]). In 2004, Pucci et al. linked cytoplasmic CLU to tumor progression, while Scaltriti et al. (2004[144]) showed that its translocation to the nucleus triggers apoptosis in prostate cancer cells. It is plausible to assume that an altered ratio of secreted CLU may play a role in tumor progression (Loison et al., 2006[93]). It was found that androgen response element in the first intron of CLU upregulates isoform 2, while isoform 1 remains unchanged (Cochrane et al., 2007[24]). This suggests that androgens may control the cytoprotective and antiapoptotic role of CLU in cancer progression. Yamamoto et al. (2015[186]) used a lipid nanoparticle-siRNA method to inhibit CLU increase induced by androgen receptor-antisense. This intervention reduced tumor growth and lowered serum PSA levels in enzalutamide-resistant LNCaP xenografts compared to AR-antisense alone. CLU is normally expressed at low levels in most cells (Viard et al., 1999[170]), but is strongly upregulated in response to various stress factors (Loison et al., 2006[93]). In 2009, the mechanism by which CLU prevents cell death was elucidated (Trougakos et al., 2009[167]). In particular, CLU stabilizes the interaction between Ku70 and the apoptotic protein Bax (Figure 4(Fig. 4)). This prevents Bax from triggering cell death by binding to the outer mitochondrial membrane. High levels of sCLU can promote tumor growth by destabilizing the Ku70-Bax complex. When sCLU is removed from this complex, it causes Bax to move to the mitochondria, triggering the release of cytochrome c and caspase 9 and inducing apoptosis (Trougakos et al., 2004[168], 2009[167]). Depletion of sCLU activates p53 and shifts the balance towards the proapoptotic Bcl-2 proteins, leading to mitochondrial dysfunction and apoptosis. Remarkably, the suppression of Bcl-2 occurs independently of p53 (Hemann and Lowe, 2006[59]). sCLU promotes cell survival by upregulating the phosphatidylinositol 3-kinase (PI3K)/protein kinase B (AKT) pathway. In addition, insulin-like growth factor (IGF-1) activates this pathway by increasing sCLU levels (Ma and Bai, 2012[97]). sCLU affects clear cell renal carcinomas by modulating ERK1/2 signaling and MMP-9 expression, thereby influencing tumor migration, invasion, and metastasis (Wang et al., 2014[174]). In epithelial ovarian cancer, overexpression of CLU correlates with increased angiogenesis and chemoresistance (Fu et al., 2013[49]). In prostate cancer, sCLU affects the nuclear translocation and activity of NF-κB; silencing of sCLU stabilizes IκB and reduces NF-κB gene transcription (Zoubeidi et al., 2010[194]). CLU acts as a downstream mediator of TGF-beta in prostate cancer, which is activated by Twist1 (transcription factor). This activation promotes epithelial-mesenchymal transition and favors metastasis. Suppression of CLU and Twist1 inhibits TGF-beta-induced cell proliferation (Shiota et al., 2012[151]). The pro-apoptotic form nCLU triggers apoptosis (Leskov et al., 2003[88]). In prostate cancer, nCLU downregulates cyclin B1 and CDK1, which leads to cell cycle arrest in the G2-M phase and thus to cell death (Scaltriti et al., 2004[144]). The different pathways by which CLU signaling regulates cell death and survival are well understood (Zhang et al., 2023[192]).

### Targeting CLU in cancer treatment

CLU, a stress-activated cytoprotective chaperone, is upregulated in response to cancer therapies, promoting treatment resistance. Testicular seminoma, with lower CLU expression, shows increased sensitivity to radiotherapy and chemotherapy (Tang et al., 2013[158]). Targeting CLU with small molecule inhibitors is challenging due to its complex posttranslational processing. Silencing CLU at the gene expression level using siRNA or antisense methods enhances chemosensitivity to various drugs, including interfering RNA with genotoxic agents (Trougakos et al., 2004[168]), tamoxifen treatment in breast cancer (Redondo et al., 2007[134]), cisplastin in renal cell carcinoma (Lee et al., 2002[84]), gemcitabine in pancreatic cancer cells (Xu et al., 2015[184]), cisplatin efficacy increased by reducing AKT and ERK1/2 phosphorylation in lung cancer cells both *in vitro* and *in vivo* (Zhang et al., 2014[189]). CLU silencing affects both isoforms. Additionally, melittin, a traditional Chinese medicine, decreased gemcitabine resistance in pancreatic ductal adenocarcinoma by targeting CLU (Wang et al., 2017[175]). In contrast, there was increased paclitaxel resistance in ovarian cancer cells with high CLU levels. This resistance was due to CLU binding to paclitaxel, obstructing its interaction with microtubules and impeding apoptosis induction (Park et al., 2008[121]). 

sCLU, identified as a difficult druggable target, requires intervention at the mRNA level. OGX-011, an antisense inhibitor, targets the initiation site (ATG site) of human exon 2 of CLU and inhibits translation (Miyake et al., 2005[106]). OGX-011 (custirsen), a second-generation antisense oligonucleotide, has a tissue half-life of ~7 days and effectively suppresses sCLU levels *in vitro* and *in vivo*. It has improved efficacy in preclinical models of prostate, lung, kidney, and breast cancer, enhancing sensitivity to chemotherapy, radiation, and hormone deprivation. Custirsen showed promising results in phase I and II prostate cancer trials (Chi et al., 2005[20], 2010[22]). However, the phase III trial showed no significant survival improvement. Adding custirsen to docetaxel and prednisone was well tolerated but did not significantly enhance overall survival for metastatic castration-resistant prostate cancer patients (Chi et al., 2017[21]). Similarly, OGX0-11 in combination with gemcitabine and platinum in stage IIIB/IV non lung cancer (NSCLC) showed some levels of higher survival rate to some extent (Laskin et al., 2012[82]) and another large phase III trial is needed. A phase II trial of custirsen with docetaxel in metastatic breast cancer showed some clinical relevance. Further studies are needed to confirm the efficacy of custirsen (OGX-011) across various cancers for personalized therapies. AB-16B5, a humanized monoclonal antibody against sCLU, was recently developed to investigate its pharmacokinetics and dynamics in patients with advanced malignancies (Ferrario et al., 2017[42]). 

**Figure 5:**
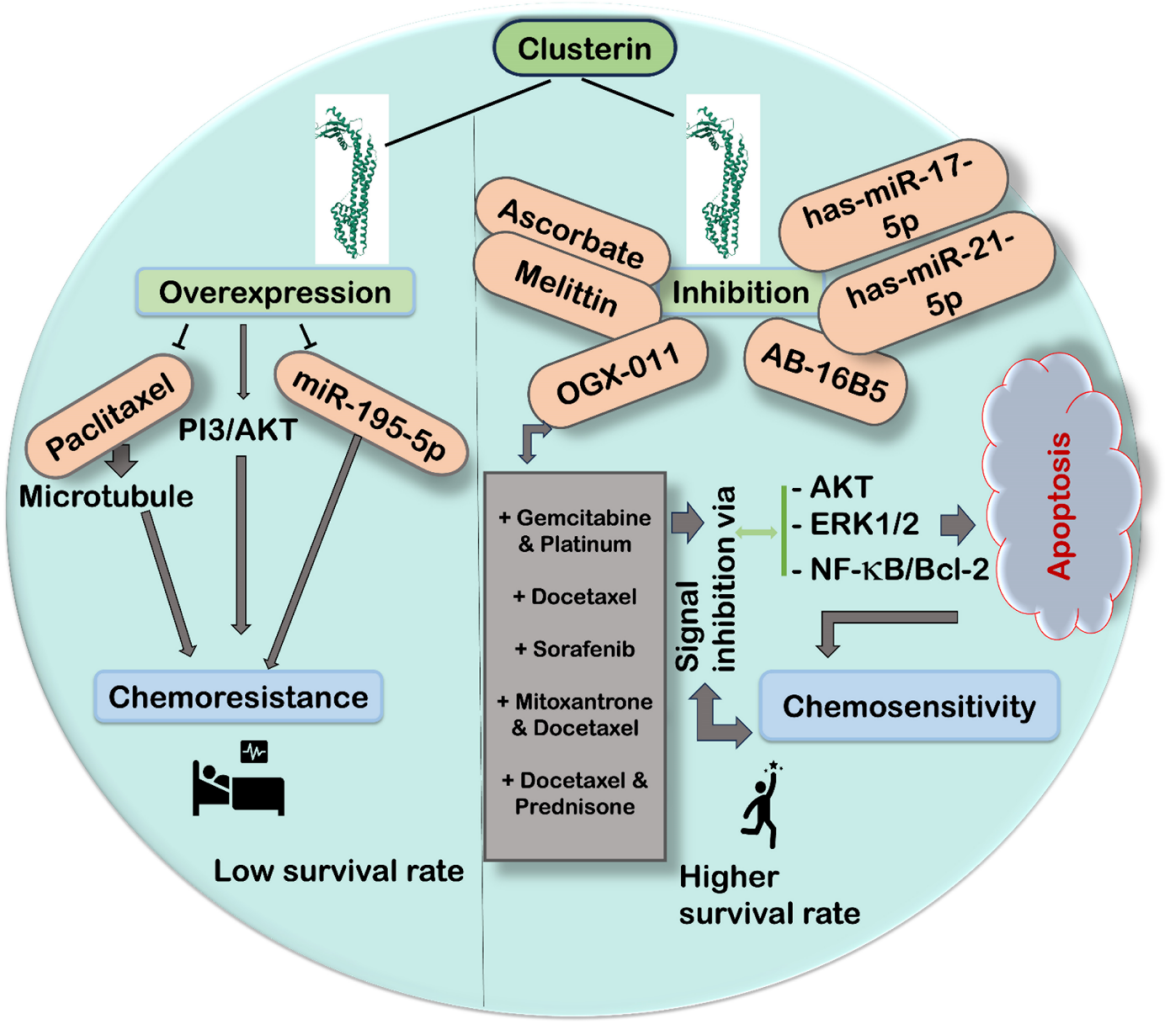
Schematic representation of the effects of clusterin overexpression on cell survival and its apoptosis induced by CLU inhibition. The figure shows that increased CLU expression correlates with decreased survival, possibly mediated by activation of survival pathways such as AKT, ERK-1/2, NF-κB, and upregulation of anti-apoptotic Bcl-2. Conversely, inhibition of CLU expression leads to apoptosis, as evidenced by downregulation of these pathways, promoting cell death. The interplay between clusterin and key signaling pathways is shown, highlighting the dual role of clusterin in regulating cell fate.

Given the unsuccessful results observed in the phase III trials, more effective approaches are needed to target CLU regulators. For example, inhibition of IGF-1R (insulin like growth factor-1 receptor) downregulates sCLU (Criswell et al., 2005[28]), ascorbate suppresses CLU and induces apoptosis in melanoma cells (Mustafi et al., 2017[110]), and, finally, miR-378 suppresses CLU and increases chemosensitization in non-small cell lung cancer (Chen et al., 2016[19]) (Figure 5(Fig. 5)).

## Clusterin in Senescence

**Figure 6:**
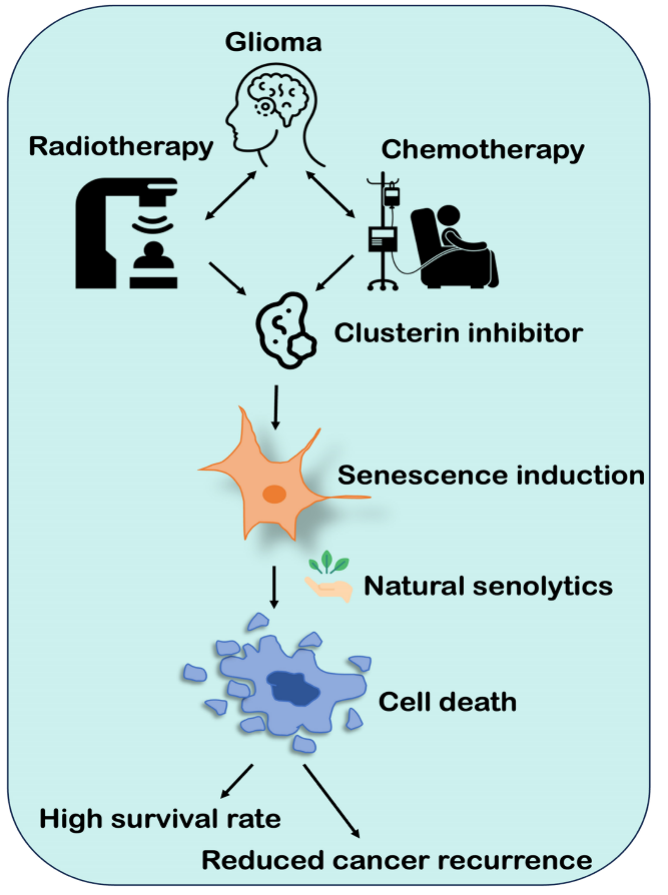
Illustration showing the role of clusterin in gliomas and its possible therapeutic modulation. The figure illustrates the influence of CLU on glioma cells and emphasizes its importance for cell survival. In addition, it highlights the strategy of inducing senescence in glioma cells by CLU inhibition and senolytic treatment is empasized, leading to a significant increase in patient survival. The scheme aims to illustrate the complex interplay between CLU, glioma progression and therapeutic interventions, emphasizing the promising approach of combining CLU inhibition with senolytics to improve clinical outcomes in glioma patients.

Cellular senescence is a phenomenon associated with irreversible cell cycle arrest mediated by increased expression of inhibitors of cyclin-dependent kinases (CDK), in particular CDKN2A/p16Ink4a and CDKN1A/ p21CIP1/WAF1 (Cohen and Torres, 2019[25]). This arrest triggers remarkable changes in cell morphology, including an increase in cell volume and lysosomal activity, and is accompanied by increased senescence-associated β-galactosidase (SA-β-gal) activity (Dimri et al., 1995[39]). This phenomenon is important for developmental processes, tissue regeneration, aging (Calcinotto et al., 2019[12]) and age-related diseases as well as cancer (Parrinello et al., 2005[123]). Recently, understanding and harnessing senescence to improve cancer treatment has become a significant challenge in the field (Foulkes and Sharpless, 2021[46]). Senescence has a significant impact on tumor biology as it exhibits both tumor suppressive and tumor promoting effects and also influences response to treatment (Schmitt et al., 2022[145]). This delicate relationship has gained attention when considering the appropriate treatment of brain tumors such as gliomas (Fletcher-Sananikone et al., 2021[45]). Gliomas account for about 80 % of all malignant brain tumors, with GMB being one of the most aggressive brain tumors, killing patients within two years (Ostrom et al., 2021[119]). Current standard treatments include surgical removal, radiotherapy, and chemotherapy, such as temozolomide, which inhibits the growth of the glioma by inducing senescence. However, a significant proportion of patients experience tumor recurrence due to dormant senescent cancer cells that are subsequently activated (Puig et al., 2018[129]; Stupp et al., 2005[154]). Given the detrimental consequences of prolonged accumulation of senescent cells and their potential role in tumor recurrence, it may be critical to use a combination of therapies to target and effectively eliminate these cancer cells. The recently coined term “one-two punch” therapy could be considered a breakthrough approach to eradicating cancer cells (Wang et al., 2017[172]). This approach is based on induction of senescence by radio- and chemotherapy, followed by potent senolytics that specifically eradicate the senescent cells by inducing apoptosis through targeting pro-survival proteins of the Bcl-2 family (Schwarzenbach et al., 2021[147]) (Figure 6(Fig. 6)). 

As mentioned above, targeting the overexpression of CLU in various cancers has the potential for tumor suppression. A study on pancreatic cancer has shown that targeting CLU with RNA induces senescence in these cells (Mitsufuji et al., 2022[104]). Although the exact pathway is still unclear, this report suggests the involvement of DNA damage, as the DNA damage marker yH2AX is upregulated. In addition, melittin, a substance derived from traditional Chinese medicine, has been identified as a potential inhibitor of tumor growth in pancreatic ductal adenocarcinoma cells by targeting the cholesterol synthesis pathway, in which CLU plays an important role in preventing apoptosis (Wang et al., 2017[175]). Both treatment with melittin and silencing of CLU can apparently significantly inhibit cell proliferation. In particular, combined inhibition of CLU together with melittin treatment has shown the ability to suppress the NF-κB/Bcl-2 and ERK signaling pathways (Wang et al., 2017[175]). These results highlight the therapeutic potential of melittin in cancer treatment, especially when used in conjunction with strategies targeting specific molecular pathways critical for tumor progression. Further research is needed to elucidate the intricate mechanisms underlying the anti-tumor effects of clinical applicability of this approach. In support of these findings, knockdown of CLU has been found to be critical for gemcitabine-induced apoptosis in human pancreatic cancer cells, which involves downregulation of NF-κB and Bcl-2. Conversely, activation of CLU promotes resistance through NF-κB-mediated transactivation and upregulation of Bcl-2 (Xu et al., 2015[184]).

It is clear now that upregulation of CLU in various cancer cells plays a role in tumor progression. This involvement occurs through the activation of several signaling pathways, such as the AKT pathway, and the promotion of MMP expression, particularly in hepatocellular carcinoma cells (Wang et al., 2015[171]; Mitsufuji et al., 2022[104]). Activation of p38 kinase (mitogen-activated protein kinase p38) plays a crucial role in the induction of senescence, albeit indirectly, as it responds to various stress stimuli (Iwasa et al., 2003[65]). Interestingly, no significant deviation was observed in the phosphorylation status of Akt, p38 or ERK proteins in pancreatic cancer cells. This observation prompts further investigation as to whether similar trends can be observed in other types of cancer cells. Therefore, conducting meaningful studies is essential to determine the involvement of these MAPKs in the process. The lack of studies investigating the mechanism by which CLU induces senescence in different cancer cell types is a significant limitation.

In the context of gliomas, a 2005 gene expression profiling study conducted revealed an increase in CLU levels in glioblastoma patients (Dong et al., 2005[40]). Based on the above report, this approach could potentially be explored as a means of inducing senescence in glioma cells. Recently, we observed that silencing of CLU can induce cell cycle arrest in astrocytoma cells and induce cellular senescence (unpublished data). 

## Clusterin in Neurodisorders

### Alzheimer's disease

Alzheimer's disease (AD) is the most common neurodegenerative disorder, accounting for more than 60 % of the 48.6 million reported cases worldwide. Given the rapid increase in the aging population, an increase to 130 million cases is predicted by 2050 (Prince et al., 2015[127]). Within the Alzheimner´s spectrum, 32 million people are affected by dementia, 69 million people by AD in the prodromal stage and 319 million by AD in the preclinical stage. Together, these stages comprise 416 million people across the AD continuum, representing 22 % of the population aged 50 years and older (Gustavsson et al., 2023[54]). The basic features of AD pathology include the presence of amyloid plaques and neurofibrillary tangles (NFTs). Amyloid plaques, extracellular aggregations of abnormally folded Aβ peptides, predominantly comprise Aβ42. The amyloid theory states that the primary pathology involves the accumulation of Aβ, resulting from cleavage of the amyloid precursor protein (APP) by the enzymes β- and γ-secretase. The key mechanism underlying Alzheimer's pathology revolves around the imbalance between the production and clearance of Aβ, emphasizing the importance of aberrations in this process (Hardy and Selkoe, 2002[57]).

Astrocytes play a crucial role in maintaining brain homeostasis by supporting neuronal function and survival. They also regulate inflammation in the central nervous system (CNS) and are associated with neurological disorders (Serrano-Pozo et al., 2011[149]). Reactive astrocytes located in close proximity to amyloid plaques, are thought to facilitate the clearance of aggregates by endocytosis (Ries and Sastre, 2016[136]). Astrocytes predominantly produce CLU, and this CLU contributes to the removal of Aβ aggregates through binding and endocytosis (Wyatt et al., 2011[182]). However, the exact mechanism of this process remains unclear. Research has shown that CLU interacts with Aβ, interfering with its fibril formation and altering Aβ-induced neurotoxicity. CLU plays a role in the transport Aβ and facilitates its clearance across the blood-brain barrier via the megalin/LRP-2 receptor pathway (Hammad et al., 1997[55]; Mulder et al., 2014[108]). Furthermore, studies have revealed that CLU can attenuate the toxicity of Aβ oligomers in *C. elegans* models (Beeg et al., 2016[7]). CLU has the ability to bind to and break down oligomers formed during the aggregation and disassembly of Aβ1-40 monomers. This action inhibits the ongoing aggregation or dissociation of these oligomeric species (Narayan et al., 2011[113]). Follow-up studies with apoE−/−/clusterin−/− double knockout mice demonstrated the synergistic effect of CLU and ApoE in reducing Aβ levels and deposition in the brain (DeMattos et al., 2004[35]). In contrast, numerous other studies have reported an opposite result, suggesting that CLU may actually impede Aβ clearance and promote Aβ aggregation, leading to neurotoxicity. Thus, preliminary experiments in PC12 cells and organotypic mouse brain slices have shown that CLU can enhance the toxicity of Aβ towards neurons and stimulate the continued aggregation of Aβ to oligomers (Oda et al., 1995[116]). Moreover, there is some evidence that CLU-dependent induction of Dickkopf-1 (DKK1) expression mediates Aβ-induced neurotoxicity. This induction also activates the Wnt-planar cell polarity-JNK signaling pathway, which involves crucial genes such as EGR1 (early growth response 1), NAB2 (Ngfi-A binding protein 2), and KLF10 (Krüppel-like factor 10), all of which play essential roles in Aβ neurotoxicity (Killick et al., 2014[75]). Ultimately, one study uncovered the debate surrounding the role of CLU (Yerbury et al., 2007[188]). It has been suggested that the nature of the interaction between Aβ and CLU depends on the CLU:Aβ ratio and the dominancy of either one may dictate whether CLU exhibits neuroprotective or neurotoxic properties.

### Parkinson's disease

Parkinson's disease (PD), the second most common progressive neurodegenerative disorder in older Americans, is expected to increase with the aging population. It results from the pathophysiologic loss of dopaminergic neurons in the substantia nigra of the midbrain and the development of neuronal Lewy bodies. Associated risk factors include age, family history, exposure to pesticides and environmental chemicals. The ultimate cause remains unknown. Clinically, the disease manifests with motor symptoms (resting tremor, rigidity, bradykinesia, and stooped posture) and non-motor symptoms, including neurobehavioral disturbances, cognitive impairment, and autonomic dysfunction (Kalia and Lang, 2015[74]).

As already mentioned, the upregulation of CLU is apparently an important risk factor in neurodegenerative disordes. In PD, CLU is mainly found in the Lewy bodies and promotes the formation of α-synuclein through its chaperone property (Sasaki et al., 2002[142]). The familial and sporadic development of PD is associated with a malfunction of the ubiquitin-proteasome system. In neuronal cells, impairment of the proteasome leads to an increase in sCLU, suggesting a link between CLU and Lewy bodies in PD as CLU prevents aggregation of α-synuclein by binding this protein present in Lewy bodies (Carreras et al., 2005[14]). Proteomic studies revealed higher levels of CLU in CSF and plasma of PD patients (Maarouf et al., 2012[99]). In fact, the association of CLU was much stronger in PD dementia than in PD alone (Gao et al., 2011[50]). Another proteomic study showed a significant increase of CLU in the serum of PD patients (Zhang et al., 2012[191]). A single-molecule fluorescence study revealed the neuroprotective function of CLU through its direct interaction with α-synuclein, shielding the α-oligomer and preventing its passage across the lipid membrane, thereby preventing the induction of reactive oxygen species (ROS) production and α-synuclein-induced toxicity (Whiten et al., 2018[177]). The protective role of CLU was supported by another study, in which downregulation of CLU led to an increase in α-synuclein without affecting cell viability (Lenzi et al., 2020[86]). Aggregation of α-synuclein oligomers leads to conformational changes that form more stable complexes. These structural changes play an important role in the subsequent fibrillization process and make toxic deposits less susceptible to degradation (Whiten et al., 2018[177]). CLU, which has been identified as a "holdase", has the ability to prevent aggregation and precipitation of protein deposits, but is unable to refold misfolded proteins (Chaplot et al., 2020[17]). CLU has also been found to be effective in preventing the aggregation process of various proteins, including α-synuclein (Yerbury et al., 2007[188]). Binding between CLU and α-synuclein has been reported to occur via regions of exposed hydrophobicity on the surface of the client protein. This study emphasized the specificity of this binding, which occurs through hydrophobic interactions and is inhibited by bisANS, a probe for solvent-exposed hydrophobic regions (Whiten et al., 2018[177]). Each chaperone has a specific domain that is critical for binding a client protein. In the case of CLU, this domain, which is functionally related to α-crystallin, is thought to be located between residues 286 and 343 (Wilson and Easterbrook-Smith, 2000[179]). In the early stages, CLU can exert beneficial effects. However, in the later, more advanced stages, which are characterized by a significant accumulation of toxic α-synuclein aggregates, the influence of CLU can potentially trigger neurodegeneration by binding to and limiting its uptake by human and murine astrocytes (Filippini et al., 2021[43]).

### Huntington's disease

Huntington's disease (HD) is an inherited neurodegenerative disorder characterized by neuropsychiatric symptoms, often choreiform movement patterns, and gradual cognitive deterioration. Confirmation of the diagnosis is usually made by detecting an increased CAG repeat length in the huntingtin gene in individuals who exhibit clinical features of the disease. Although the diagnosis is generally straightforward, atypical presentations can be challenging, particularly in determining the transition from asymptomatic carrier to disease (Cepeda and Tong, 2018[15]). A limited number of studies have investigated the role of CLU in HD. In 1999, a study demonstrated the strong expression of CLU in the caudate nucleus of HD brain slices, highlighting its involvement in the regulation of the complement system (Singhrao et al., 1999[153]). The increased expression of CLU is also associates with various physiological and pathological conditions, including apoptosis and the response to injury. CLU has previously been linked to other neurodegenerative disorders such as AD, where its plasma levels correlate with the extent of neurodegeneration. Interestingly, CLU was detected in both peripheral plasma and cerebrospinal fluid, indicating widespread immune activation. Proteomic profiling indicated that CSF from HD patients contained increased CLU amounts (Dalrymple et al., 2007[30]). It is not clear how CLU may influence pathology of HD and further research is needed to understand the mechanism.

### Amyotrophic lateral sclerosis

Amyotrophic lateral sclerosis (ALS) is a progressive neurodegenerative disease characterized by muscle wasting that leads to paralysis and eventually death (Goldstein and Abrahams, 2013[51]). Cognitive dysfunction often occurs simultaneously in ALS patients. A pathological hallmark of ALS is the presence of a 43 kDa protein known as Tar-DNA binding protein (TDP-43), which exhibits misfolded accumulation in the brain and spinal cord of ALS patients (Neumann et al., 2006[114]). Mass spectrometry analysis showed significantly increased expression of CLU in the ALS group with cognitive impairment compared to healthy controls (Xu et al., 2018[185]). According to a recent study, CLU may act as a protective shield in ALS. It could achieve this by retrotranslocating TDP-43 from extracellular to intracellular locations, thus preventing the formation of misfolded aggregates (Gregory et al., 2020[52]). In a recent ELISA-based study, significantly elevated CLU levels in the cerebrospinal fluid of ALS patients indicated that CLU is a potential diagnostic marker for neurodegenerative diseases (Klíčová et al., 2024[77]). However, differences between disease stages and individual patients may affect the discriminatory ability.

### Traumatic brain injury

Traumatic brain injury (TBI) and stroke significantly increase CLU mRNA levels and CLU immunoreactivity in neuronal and astroglial subpopulations (Bellander et al., 2001[8]). This CLU production is accompanied by local synthesis of complement components by microglia/macrophages (Bellander et al., 2001[8]). Activation of the complement cascade and upregulation of CLU have also been observed in rat models of sciatic nerve and optic nerve injury (Liu et al., 1995[90]; Ohlsson et al., 2003[118]) and spinal cord trauma (Anderson et al., 2005[2]). In the traumatically brain-injured rats, a significant increase in extracellular CLU (7 days to 1 month post-TBI) was observed in the perilesional cortex as well as in the ipsilateral hippocampus and ipsilateral thalamus (Das Gupta et al., 2019[32]). A differential increase in CLU concentration in samples from patients with TBI was associated with longer survival. This suggests that CLU acts as a modulator of the inflammatory response in acute and chronic TBI (Troakes et al., 2017[165]). Significant accumulation of CLU in astrocytes and increased inflammation and microglial activation suggest a role for this protein in TBI-related neurodegenerative changes. Long-term expression of CLU in astrocytes after ischemia may contribute to enhanced brain tissue remodeling (Imhof et al., 2006[63]). In addition to its protective role, CLU may also contribute to neuronal process formation and plasticity (Wicher et al., 2008[178]). The molecular pathways underlying the potential protective role of sCLU in brain ischemia remain largely speculative. It is widely recognized that activation of the complement cascade is deleterious in the early stages and involves neuroinflammation, formation of the membrane attack complex, heme toxicity, and free radical release. However, it later transitions to a beneficial phase, involving opsonization and clearance of apoptotic cells (Komotar et al., 2008[79]; Arumugam et al., 2009[4]; Ten et al., 2010[160]). Other mechanisms involved in the protective effect of CLU are due to the interaction of CLU and the cytokine TGF-β (Jin and Howe, 1999[69]). The involvement of TGF-β1 and TGF-β receptors in neuronal cell survival after brain ischemia (Henrich-Noack et al., 1996[60]) suggests that CLU plays a role in neuronal rescue via interaction with TGF-β.

### Retinopathies

There is evidence that different mammalian species express CLU in ocular tissues, including the ciliary body, cornea, lens, retina, and vitreous (Reeder et al., 1995[135]). Interestingly, a study in transgenic mice has shown that overexpression of CLU in photoreceptor cells protects the retina from light-induced degeneration (Jomary et al., 1999[70]). Depletion of CLU in ocular surface epithelia, which occurs in various inflammatory conditions in humans and mice, leads to squamous metaplasia and keratinized epithelium, suggesting a role for CLU in maintaining mucosal epithelial differentiation. Therefore, CLU could be a potential therapeutic target for dry eye disease (Fini et al., 2016[44]).

## Clusterin in Aging

The aging phenomenon is characterized by a gradual decline in the physiological functions of living organisms over time, due to a time-dependent deterioration. With advances in medicine enabling longer life expectancy, the economic and social impact of aging and age-related diseases has made the study of the underlying cellular mechanisms of aging a topic of great research interest. The results of clinical trials on the treatment of age-related neurodegenerative diseases have been suboptimal, leading to the hypothesis that aging not only increases the risk of disease but may also be the cause of it (Cummings et al., 2014[29]). The central nervous system undergoes several detrimental changes during aging, including mitochondrial dysfunction, oxidative stress, and chronic inflammation (Chakrabarti et al., 2011[16]). Therefore, targeting these changes in the central nervous system may have therapeutic potential to alleviate the effects of aging.

The hallmarks of aging include genomic instability, epigenetic alterations, telomere attrition, loss of proteostasis, impaired nutrient sensing, mitochondrial dysfunction, stem cell exhaustion, altered intercellular communication and cellular senescence (López-Otín et al., 2013[95]). Aging brings a multifaceted aspect to the intricate role of CLU, a versatile protein that has both deleterious effects in cancer and beneficial properties in neurological disorders. The controversial role of CLU unfolds a complex narrative that portrays it as a double-edged sword with seemingly contradictory functions in cancer and neurodegenerative disorders. In cancer, CLU has emerged as an aggressive player that promotes tumor growth, inhibits apoptosis and confers resistance to therapies. Its overexpression in various cancers raises the question of its potential as a therapeutic target for inducing senescence and preventing tumor progression. There are few studies investigating the properties of CLU in brain tumors. In this context, different approaches could be used to prevent the synthesis of the CLU protein. These strategies may include the screening of FDA-approved drugs and the use of existing inhibitors, such as the structure of OXG-011, to identify analogous drug molecules. In additional, exploration of natural senolytics such as fisetin and quercetin could be considered as a potential pathway to inhibit CLU formation (Lagoumtzi and Chondrogianni, 2021[81]). Conversely, CLU exhibits a paradoxical duality in the context of neurodegenerative disorders - it acts as both a protector and a potential source of toxicity. Studies suggest that CLU may play a neuroprotective role by preventing the aggregation of proteins such as amyloid-beta and alpha-synuclein, which play a key role in Alzheimer's and Parkinson's disease, respectively. However, this protective function comes with a caveat, as CLU interactions with these proteins can also contribute to neurotoxic effects under certain conditions.

## Epigenetic Regulation of Clusterin

Epigenetics refer to heritable changes in gene expression, passed through mitosis and meiosis that do not alter the DNA sequence itself but are crucial for regulating gene expression by influencing DNA accessibility and chromatin structure (Chuang and Jones, 2007[23]). Epigenetic regulation is also found to be linked with aging and age-related diseases (Calvanese et al., 2009[13]). CLU is typically overexpressed during aging and cancer progression. Given that both aging and cancer impact DNA methylation and histone acetylation statuses, it has been proposed that epigenetic regulation plays a significant role in modulating CLU expression as studied in retinal pigment epithelial cells (Suuronen et al., 2007[156]). CLU expression during cell transformation is modulated and increases in gliomas (Danik et al., 1991[31]). The promoter region of CLU contains binding sites for transcription factors and a CpG-rich methylation domain (Suuronen et al., 2007[156]). Irregular epigenetic regulation of CpG islands may impact the risk of developing integrated late onset AD (Wang et al., 2008[173]). Some studies suggest that epigenetic factors could also influence amyloidogenesis in AD (Wu et al., 2008[181]). Importantly, a higher plasma concentration of CLU in AD patients seems to be positively associated with fibrillar Aβ burden (Thambisetty et al., 2010[162]). Since CLU is highly modulated in diverse pathological processes, it is important to investigate how its dynamic function can be influenced by epigenetic regulation in cancer and neurodegenerative diseases. In cancer, alteration of DNA methylation and histone acetylation patterns can inhibit CLU, leading to cellular senescence and disrupting tumor growth. Conversely, epigenetic changes in neurological diseases can enhance the neuroprotective properties of CLU, offering potential therapeutic benefits in conditions such as AD. Understanding these regulatory mechanisms is critical for the development of targeted therapies that address the distinct roles of CLU in these diseases.

### Histone modifications 

Histone proteins are located in the core of the chromatin framework, where they regulate the compaction and decompaction of DNA. The diversity of histone post-translational modifications dictates different chromatin configurations, each of which fulfills distinct functions, including the maintenance of genome stability (Allis and Jenuwein, 2016[1]). Histones undergo post-translational modifications such as methylation, acetylation, phosphorylation, ubiquitination, glycosylation and many more (Tessarz and Kouzarides, 2014[161]). Such modifications affect the gene expression of almost all genes. These alterations influence how histones interact with DNA and impact the transcription process (Jenuwein and Allis, 2001[67]). In line with the Histone Code Hypothesis, various combinations of histone modifications control both chromatin structure and transcriptional activity. Tightly bound histones generally suppress gene expression, while the positively charged amino acids of modified histones support the recruitment of transcription factors to specific DNA sequences (Cosgrove and Wolberger, 2005[27]). It is unclear how the terminal end of histones influences the interaction between DNA and histones, but it has a major impact on chromatin structure. One known specific motif in particular is the modified histone 3 (H3). Acetylation (H3K9ac) at the tails can lead to a loss of interaction with DNA and access to transcription factors (Roth et al., 2001[139]). On the other hand, methylated histones can modulate transcription differently. The enrichment in histone H3 lysine 9 trimethylation (H3K9me3) and histone H3 lysine 27 trimethylation (H3K27me) leads to the downregulation of nCLU, while the increase in the histone H3 lysine 4 trimethylation (H3K4me3) or histone H3 lysine 9 acetylation and serine10 phosphorylation (H3K9Acs10P) causes activation of nCLU, which leads to cell death (Deb et al., 2015[34]). In prostate cancer cells, treatment with epigenetic drugs enhances histone H3 lysine 4 trimethylation (H3K4me3), decreases histone H3 lysine 27 trimethylation (H3K27me3), and induces CLU1 and CLU2 transcription. The di- and tri-methylation of histones mediates transcription factors to specific DNA regions. CLU may thus react to epigenetic drug treatment via histone modification (Bonacini et al., 2015[11]). This type of response has been observed in different cancer cells (Bonacini et al., 2015[11]; Hellebrekers et al., 2007[58]), in which the use of DNA methyltransferase inhibitors and histone deacetylases increases CLU expression. In neuroblastoma cells, high MYCN recruited by HDACs (histone deacetylases) suppresses the CLU expression (Corvetta et al., 2013[26]). In contrast, CLU expression before the onset of AD can simultaneously reduce the risk of developing the disease. A potential modulator of interest is age-related changes in sex hormones, as CLU isoforms are differentially regulated by androgens (Cochrane et al., 2007[24]). Therapeutic agents such as valproate act as histone deacetylase inhibitors and increase CLU expression in human astrocytes (Nuutinen et al., 2010[115]). Interestingly, HDAC inhibitors also regulate CLU in retinal pigment epithelial cells and influence the pathogenesis of age-related macular degeneration by inhibiting angiogenesis and inflammation (Suuronen et al., 2007[156]). Increasing CLU expression helps to reduce amyloid accumulation and behavioral deficits in mouse models of amyloidosis (Qing et al., 2008[131]), suggesting a protective mechanism against AD risk. 

### DNA methylation 

In addition to histone modifications, DNA methylation contributes to the repression of transcription in differentiated mammalian cells. In this process, methyl groups are added to the C5 position of cytosine in CpG islands, which alters transcription without changing the DNA sequence (Zhang et al., 2020[190]). The GC-rich region with methylated CpG island located just upstream the 5' end of the CLU promoter region indicates that DNA methylation regulates CLU gene expression (Serrano et al., 2009[148]). Reduced CLU expression due to methylation of the promoter was found in breast cancer (Serrano et al., 2009[148]), ovarian epithelial cancer (Yang et al., 2013[187]) and human prostate carcinoma (Rauhala et al., 2008[133]). However, demethylation of the promoter using epigenetic drugs in endothelial cells (Hellebrekers et al., 2007[58]) and retinal pigment epithelial cells (Suuronen et al., 2007[156]) significantly increased CLU gene expression. This suggests that CLU is regulated differently in different cancer cell types and that hypo- or hypermethylation leads to low or high CLU gene expression and different levels of sCLU and nCLU isoforms (Martindale and Holbrook, 2002[101]). Further studies in rats have shown that hypomethylated CLU is present in tissues in which CLU is consistently expressed, but not in tissues with lower expression of CLU (Rosemblit and Chen, 1994[138]). CLU expression may therefore vary in unmethylated tumors. Nevertheless, methylation affects only a small fraction of CpG dinucleotides and represses the transcription of a specific subset of genes in differentiated cells and plays a crucial role in gene suppression during differentiation (Handy et al., 2011[56]). DNA methylation is of great interest in AD due to its age-related alterations (Wang et al., 2018[176]). A study conducted on AD patients in Japan found that they had reduced DNA methylation of the CLU gene, which is considered an independent risk factor for the onset of AD. Furthermore, hypomethylation of the CLU gene was observed in the blood of patients suffering from dementia with Lewy bodies. These findings suggest that hypomethylation of the CLU gene could be disease-specific in neurodisorders (Mitsumori et al., 2020[105]).

### MicroRNAs 

MicroRNAs (miRNAs) are short, non-coding RNAs that typically comprise around 22 nucleotides. They play a crucial role in the regulation of gene expression by inducing post-transcriptional modifications of mRNA transcripts and thereby influence various cellular processes (Bartel, 2004[6]). Irregular expressions of miRNAs, which act either as tumor suppressors or oncogenes, are closely linked to the prognosis of numerous types of cancer (Iorio and Croce, 2012[64]).

Based on previous studies, it is obvious that CLU is regulated by miRNAs. For example, CLU was identified as a specific target of the oncogenic miRNA-21 in head and neck squamous cell carcinomas. In this case, the CLU1 isoform was downregulated, which is a growth-suppressive variant (Mydlarz et al., 2014[111]). In prostate cancer, miR-195 was found to increase the sensitivity of resistant prostate cancer to docetaxel by inhibiting CLU expression (Ma et al., 2018[98]). In addition, miR-217-5p can regulate CLU by inhibiting EMT-related proteins, thereby limiting prostate cancer cell invasion and migration (Zhao et al., 2021[193]). In lung adenocarcinoma, miRNA-378 targets sCLU to suppress cell growth by reversing chemoresistance to cisplatin (Chen et al., 2016[19]). Interestingly, CLU is downregulated in neuroblastoma. Here, CLU expression is apparently suppressed by the miR-17-92 cluster induced by the proto-oncogene MYCN (Chayka et al., 2009[18]). Furthermore, the expression of sCLU was found to be markedly reduced in the peripheral blood plasma of pregnant women with abnormally invasive placenta, while the levels of its regulatory microRNAs hsa-miR-17-5p, hsa-miR-21-5p, hsa-miR-25-3p, hsa-miR-92a-3p and hsa-miR-320a-3p were significantly increased (Timofeeva et al., 2021[164]). The controversial role of CLU suggests that CLU expression in various cancers is context- and signaling-dependent, with the ratio of sCLU to nCLU determining cell survival or death. 

### Transcription factors

Basal transcription and its regulation depend on transcription factors that bind to specific DNA sequences in gene regulatory regions to control transcription. These factors are classified into families based on their protein structures that enable DNA binding or factor dimerization. This is therefore an important first step in gene expression, followed by post-transcriptional processes such as RNA splicing and translation (Latchman, 1993[83]). 

A recent study using electrophoretic mobility shift assays showed that expression of CLU is regulated by a basal promoter and two more distal negative regulatory regions (Gross et al., 2021[53]). A specific example of transcription factors regulating CLU expression are caudal-related homeobox gene transcription factors during intestinal development (Suh et al., 2001[155]). There is also evidence that CLU expression is influenced by transcription factors encoded by oncogenes. In the 1980s, the induction of the T64 gene by a retroviral oncogene with protein kinase activity was reported in avian cells (Michel et al., 1989[103]). Later, T64 was identified as the avian homolog of rat CLU, whose functionality is closely linked to the presence of the AP-1 binding motif within the CLU transcription initiation region (Herault et al., 1992[61]). TGFβ has been demonstrated to enhance CLU expression by activating an AP-1 site within the mammalian CLU promoter. This activation occurs via the abrogation of the trans-repression effect of c-Fos by TGFβ (Jin and Howe, 1999[69]). TGF-β was observed to upregulate CLU in rat astrocytes co-cultured with microglia and oligodendrocytes (Morgan et al., 1995[107]), with CLU promoting neuronal cell survival during brain ischemia. The observation showing that exposure of HaCat keratinocyte cells to vanadium induces apoptosis via c-Fos expression by switching sCLU to nCLU (Markopoulou et al., 2009[100]) suggests that c-Fos affects the ratio of sCLU to nCLU. It is unclear how c-Fos suppression occurs and modulates CLU expression. 

The classical proto-oncogenes c-MYC and Ha-RAS have been reported to influence CLU expression. Specifically, overexpression of Ha-RAS resulted in a reduction of CLU mRNA levels in rat embryo fibroblasts (Klock et al., 1998[78]), RAS has been shown to induce CLU promoter deacetylation, followed by methylation of the CpG island leading to CLU suppression (Lund et al., 2006[96]). Ectopic levels of c-MYC significantly reduced CLU expression in murine colonocytes, inhibiting the growth *in vitro* and carcinogenesis *in vivo* (Thomas-Tikhonenko et al., 2004[163]). c-MYC plays a pivotal role in human tumorigenesis, and its function in cancer has been extensively reviewed in numerous studies (Dhanasekaran et al., 2022[38]). Neuronal MYC (MYCN) is a negative regulator of CLU and was found to be downregulated in pediatric neuroblastoma (Chayka et al., 2009[18]). This downregulation is indirectly affected by the miR-17-92 microRNA cluster (Dews et al., 2006[37]; O'Donnell et al., 2005[117]). Mice with a disrupted CLU gene are more susceptible to developing neuroblastomas when MYCN is transgenically expressed, suggesting that the MYCN-CLU axis is crucial and that CLU acts as a repressor in tumorigenesis. MYCN can directly repress CLU transcription via the E-box located in the 5' flanking region of the CLU gene (Chayka et al., 2009[18]). In prostate cancer cells, the Twist transcription factor binds to the E-box in the CLU gene and mediates TGFβ-induced CLU expression. The Twist-CLU pathway apparently serves as a significant mediator in TGFβ-induced epithelial-mesenchymal transition and cell migration and invasion (Shiota et al., 2012[151]). In addition, Twist is essential for IGF-I-mediated CLU expression and growth signaling in castration-resistant prostate cancer, as IGF-I activates the STAT3-Twist1 signaling pathway, leading to Twist1 binding to E-boxes on the CLU promoter (Takeuchi et al., 2014[157]). Modulation of CLU by these factors suggests that CLU acts as a repressor in certain cancers, while in others it is essential for growth signaling and metastasis. Another transcription factor, B-MYB, which is known to be a positive regulator of cell survival and is overexpressed in human cancers, has been shown to enhance CLU gene expression (Nakajima et al., 2008[112]; Sala and Watson, 1999[140]). 

NF-κB is a versatile transcription factor that has been found to strongly regulate CLU (Li et al., 2002[89]). Treatment of astrocytes with bacterial lipopolysaccharide, which activates NF-κB signaling, resulted in upregulation of CLU (Saura et al., 2003[143]). Interestingly, there is some evidence that CLU negatively regulates NF-κB activity by stabilizing IκB and thus can modulate the release of cytokines (Santilli et al., 2003[141]). Low CLU expression has been reported to be associated with excessive NF-κB activation and cytokine secretion in rheumatoid arthritis (Devauchelle et al., 2006[36]). However, another study suggested that CLU promotes matrix metalloproteinase-9 activity by facilitating the translocation NF-κB to the nucleus via IkBα degradation in macrophages (Shim et al., 2011[150]). Although NF-κB is known to regulate CLU and is predicted to have a response element in the CLU promoter, binding to this site has not been confirmed.

However, there are also other transcription factors that can influence the expression of CLU. It was found that IGF-1 receptor-initiated transactivation of the early growth response-1 (Egr-1) transcription factor, which binds to the CLU promoter, is required for increased sCLU expression upon irradiation (Criswell et al., 2005[28]). This suggests CLU secretion is upregulated as a protective response to cellular stress, as evidenced by accelerated cell death observed following RNAi-mediated silencing of CLU. In prostate cancer cells, the transcription factor Stat1 was found upstream of CLU and its deletion by siRNA suppressed CLU levels by about 50 % (Patterson et al., 2006[125]). It remains uncertain whether Stat-1 directly regulates CLU gene expression, but the presence of potential Stat-binding sites in the CLU promoter suggests this possibility. Other transcription factors found in prostate cancer cells involved in CLU regulation include Y-box binding protein-1 (YB-1) and hypoxia inducible factor 1α (HIF-1α) (Park et al., 2014[122]; Shiota et al., 2011[152]). Pax6, a paired-box transcription factor necessary for eye development, affects CLU expression in human corneal epithelial cells (Kitazawa et al., 2017[76]). Interestingly, Pax6 was found to induce CLU expression by binding to the CLU promoter in the Sjogren's syndrome mouse model (Liu et al., 2023[91]). Hence, CLU could potentially serve as a protective factor against epithelial damage or inflammation, highlighting its therapeutic potential in the treatment of ocular surface disorders.

## Conclusion

Targeting CLU represents a promising strategy for both cancer and neurodegenerative diseases. The ability of CLU inhibition to induce senescence in cancer cells provides a valuable therapeutic approach to interrupt tumor growth. Conversely, the complex role of CLU in neurodegenerative disorders, where it exhibits both protective and toxic properties, particularly when bound to protein aggregates such as amyloid beta and α-synuclein poses a major challenge. Epigenetic regulation is emerging as a viable strategy to modulate CLU expression in these diseases. By altering DNA methylation and histone acetylation patterns, and targeting specific transcription factors, CLU expression can be effectively up- or downregulated, thereby influencing disease progression. In cancer, such epigenetic interventions can modulate the role of CLU in cell survival and apoptosis, potentially reducing tumor growth and metastasis. In neurodegenerative diseases, strengthening the neuroprotective functions of CLU through epigenetic interventions could offer therapeutic benefits in diseases such as AD and brain ischemia. A deeper understanding of the dual nature of CLU is essential to develop new therapeutic strategies that can attenuate neurodegenerative pathologies while utilizing its senescence-inducing properties in cancer treatment. Further research is essential to unravel the complexity of CLU modulation and to explore its potential clinical applications in these distinct but interconnected pathological contexts.

## Declaration

### Competing interests

The authors have no competing interests.

### Acknowledgments

This work was supported by the Charles University Institutional Research Fund (SVV-260683). Figures 2-4(Fig. 2)(Fig. 3)(Fig. 4) were created with BioRender (Biorender.com, Toronto, Canada)

## Figures and Tables

**Figure 1:**
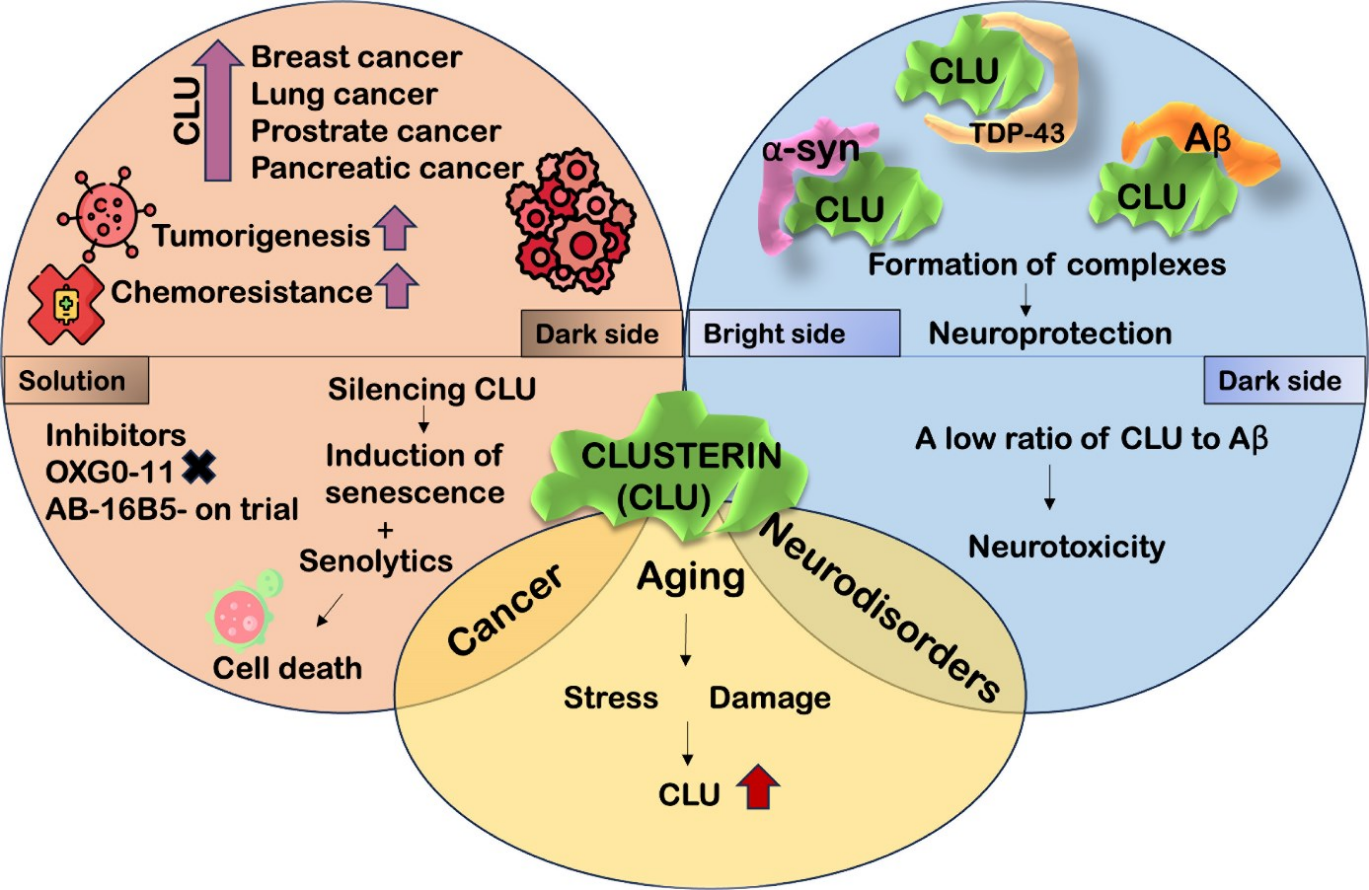
Graphical abstract
